# A first-in-man study of [^18^F] FEDAC: a novel PET tracer for the 18-kDa translocator protein

**DOI:** 10.1007/s12149-023-01895-0

**Published:** 2024-01-29

**Authors:** Kentaro Tamura, Ryuichi Nishii, Kotaro Tani, Hiroki Hashimoto, Kazunori Kawamura, Ming-Rong Zhang, Takamasa Maeda, Kana Yamazaki, Tatsuya Higashi, Masahiro Jinzaki

**Affiliations:** 1 Department of Molecular Imaging and Theranostics, Institute for Quantum Medical Science, Quantum Life and Medical Science Directorate, National Institutes for Quantum Science and Technology (QST), 4-9-1 Anagawa, Inage-Ku, Chiba, 263-8555 Japan; 2 Department of Radiology, Keio University School of Medicine, 35 Shinanomachi, Shinjuku-Ku, Tokyo, 160-8582 Japan; 3 Department of Integrated Health Sciences, Graduate School of Medicine, Nagoya University, 1-1-20 Daiko Minami, Higashi-ku, Nagoya, 461-8673 Japan; 4 Department of Radiation Measurement and Dose Assessment, Institute for Quantum Medical Science, Quantum Life and Medical Science Directorate, National Institutes for Quantum Science and Technology (QST), 4-9-1 Anagawa, Inage-ku, Chiba, 263-8555 Japan; 5 Department of Advanced Nuclear Medicine Sciences, Institute for Quantum Medical Science, Quantum Life and Medical Science Directorate, National Institutes for Quantum Science and Technology (QST), 4-9-1 Anagawa, Inage-ku, Chiba, 263-8555 Japan; 6 Department of Medical Technology, Quantum Life and Medical Science Directorate, QST Hospital, National Institutes for Quantum Science and Technology (QST), 4-9-1 Anagawa, Inage-ku, Chiba, 263-8555 Japan

**Keywords:** Translocator Protein, TSPO, Normal healthy volunteers, Positron emission tomography, Biodistribution, Radiation Dosimetry, IDAC

## Abstract

**Purpose:**

N-benzyl-N-methyl-2-[7, 8-dihydro-7-(2-[^18^F] fluoroethyl) -8-oxo-2-phenyl-9H-purin-9-yl] acetamide ([^18^F] FEDAC) is a novel positron emission tomography (PET) tracer that targets the translocator protein (TSPO; 18 kDa) in the mitochondrial outer membrane, which is known to be upregulated in various diseases such as malignant tumors, neurodegenerative diseases, and neuroinflammation. This study presents the first attempt to use [^18^F]FEDAC PET/CT and evaluate its biodistribution as well as the systemic radiation exposure to the radiotracer in humans.

**Materials and Methods:**

Seventeen whole-body [^18^F]FEDAC PET/CT (injected dose, 209.1 ± 6.2 MBq) scans with a dynamic scan of the upper abdomen were performed in seven participants. Volumes of interest were assigned to each organ, and a time–activity curve was created to evaluate the biodistribution of the radiotracer. The effective dose was calculated using IDAC-Dose 2.1.

**Results:**

Immediately after the intravenous injection, the radiotracer accumulated significantly in the liver and was subsequently excreted into the gastrointestinal tract through the biliary tract. It also showed high levels of accumulation in the kidneys, but showed minimal migration to the urinary bladder. Thus, the liver was the principal organ that eliminated [^18^F] FEDAC. Accumulation in the normal brain tissue was minimal. The effective dose estimated from biodistribution in humans was 19.47 ± 1.08 µSv/MBq, and was 3.60 mSV for 185 MBq dose.

**Conclusion:**

[^18^F]FEDAC PET/CT provided adequate image quality at an acceptable effective dose with no adverse effects. Therefore, [^18^F]FEDAC may be useful in human TSPO-PET imaging.

## Introduction

The 18-kDa translocator protein (TSPO) is expressed mainly in the outer mitochondrial membrane and was first identified as a receptor for benzodiazepines in 1977 [1, 2].TSPO is involved in fundamental cellular functions such as steroid synthesis, heme biosynthesis, cell growth, and response to oxidative stress [3]. Since increased TSPO expression occurs in various diseases such as inflammation or degeneration of the central nervous system, malignant tumors, and myocardial infarction, TSPO-specific ligands have received much attention [4–6]. Positron emission tomography (PET) scanning with a radiolabeled TSPO probe allows for noninvasive and objective assessment of TSPO expression in vivo. The first PET ligand utilized for clinical imaging of TSPO was PK11195; however, its nonspecific binding and poor signal-to-noise ratio [7, 8] have necessitated the development of new and improved PET ligands that selectively bind to TSPO [9]. Therefore, various second-generation TSPO-PET ligands were developed, including AC-5216 and [^11^C]DAC. We recently developed a novel TSPO radioligand, N-benzyl-N-methyl-2-[7, 8-dihydro-7-(2-[^18^F]fluoroethyl) -8-oxo-2-phenyl-9H-purin-9-yl] acetamide ([^18^F]FEDAC), using [^11^C]-DAC as the leading compound. It showed strong binding affinity and selectivity for TSPO. Furthermore, because it is labeled with ^18^F, it had a longer half-life and a lower energy dose than ^11^C formulations, allowing better image quality [10]. PET imaging studies using [^18^F]FEDAC in small animals have shown that it can be used for quantitative analysis with vivo imaging [11].

[^18^F]FEDAC PET imaging has been used in various animal disease models, such as neuroinflammation [12], steatohepatitis [13], atherosclerosis [14], heart failure [15], and collagen-induced arthritis [16], indicating its promise for clinical TSPO imaging. However, it has not yet been applied in humans. Therefore, in this study, whole-body PET/CT scans were performed to estimate the effective dose and the biodistribution of [^18^F] FEDAC.

## Materials and methods

### Human participants

From August to December 2021, normal healthy volunteers were recruited for this first-in-human clinical PET/CT imaging study, and seven male participants were included. The key eligibility criteria were as follows: (1) males aged 20 to 65 years and (2) BMI ≤ 25 kg/m^2^. The key exclusion criteria were as follows: (1) underlying severe disease or a history of severe disease, (2) substance dependence or alcohol addiction, and (3) administration of medical drugs within two weeks of examination. All participants received a full explanation of the clinical study with the expected radiation exposure and provided written informed consent to participate.

Participants were 40 ± 12.2 years old and ranged in age from 24–61. The mean weight of the participants was 74.0 ± 7.5 kg, mean height was 1.78 ± 0.06 m, and mean BMI was 23.4 ± 1.9.

The Certified Review Board (CRB3180004) approved this clinical trial (approval #L21-004), which was registered in the Japan Registry of Clinical Trials (jRCTs031210134). All procedures in this clinical trial were conducted in accordance with the 1964 Declaration of Helsinki and its later amendments.

### Preparation and administration of [^18^F] FEDAC

[^18^F]FEDAC was radiosynthesized in our laboratory by direct [^18^F]-fluorination of the tosylated precursor using a previously published method [17]. We planned to administer [^18^F]FEDAC intravenously at a dose of 3.7 MBq/kg ± 10%. When the subject weighed more than 60 kg, the administered radioactivity was set at 222 MBq ± 10%.

### PET/CT imaging data acquisition

The participants were required to fast for at least 5 h prior to the study. All PET/CT scans were obtained using Discovery MI (GE Healthcare, Milwaukee, WI, USA), which provides 89 sections with an axial field of view (FOV) of 25 cm. Computed tomography (CT) was performed before the emission scan for attenuation correction.

**Fig. 1:**
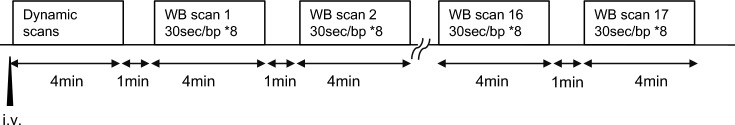
PET Scan Protocol. Dynamic scans of the upper abdomen, including the heart, were performed during the first 4 min. After 5 min, a static scan of the whole body was repeated every 5 min using 8 beds, 30 s per bed. A total of 17 whole-body scans were made

Immediately after intravenous rapid bolus injection of [^18^F]FEDAC (mean injected dose, 209.1 ± 6.2 MBq; range, 201.8–218.0 MBq/0.89 ± 0.22 μg; range, 0.66–1.28 μg), dynamic scan of the upper abdomen, including the heart, was performed for 4 min, followed by 17 static whole-body scans up to 90 min after the intravenous injection (Fig. 1).

All PET images were reconstructed in a 256 × 256 matrix using the ordered-subset expectation–maximization algorithm with time-of-flight information. Corrections for scatter and random coincidences, dead time, and attenuation were performed as provided by the camera manufacturer.

### Image analysis, biodistribution, and radiation dosimetry

PET/CT images were anonymized, stored in the DICOM format, and analyzed using PMOD software version 4.205 (PMOD Technologies Ltd., Zurich, Switzerland). All images were reviewed by board-certified diagnostic radiologists with over ten years of experience. A three-dimensional volume of interest (VOI) was placed on each organ in the PET and CT images. The semi-quantitative mean standardized uptake value (SUVmean) was used to assess the biodistribution of [^18^F]FEDAC in PET image analysis. Time–activity curves (TACs) for the first 5 min after injection of [^18^F]FEDAC were obtained by measuring the radioactive concentrations in the VOIs specified in the dynamic scan images for the following organs and tissues that showed substantial accumulation of the radiotracer in the FOV: adrenals, alveolar-interstitial region, gallbladder, heart wall, kidneys, liver, pancreas, spleen, heart content, and red marrow. The VOI of red bone marrow was established in the lumbar vertebral bodies, assuming this to represent typical red bone marrow uptake [18]. The TACs of the radiotracer from 5 to 90 min were obtained from whole-body scan images at 5-min intervals, and VOIs for the brain, thyroid, and salivary glands were specified in addition to those for the organs and tissues specified in the dynamic scan images. The volumes of the organs and tissues in the adult male phantom [19] developed by the International Commission on Radiological Protection (ICRP) were used to calculate the total radioactivity in each organ and tissue from the radioactive concentrations in the VOIs (Bq/mL). The TAC of blood was calculated by assuming that the radioactivity in the heart was 9% of the total blood radioactivity [18] and the TAC of the remaining tissue was determined so that the total radioactivity of the TACs equaled the remaining injected dose. Based on total disintegrations of [^18^F] derived from manual numerical integration of the TACs by summing the radioactivity at each time step using Microsoft Excel, organ doses and effective doses for the reference person defined in ICRP Publ. 103 were assessed using IDAC-Dose 2.1 [20, 21].

### Vital signs, blood and urine sampling, and analysis

Before and after the examination, we measured vital signs including blood pressure, pulse, and body temperature. Additionally, blood and urine tests were conducted before and after the examination. The details of these tests are as follows:

Blood tests: complete blood count, glucose, HbA1c, total bilirubin, AST, ALT, ALP, LDH, BUN, creatinine, total protein, and albumin.

Urine tests: glucose, protein, occult blood, urobilinogen, and ketone bodies.

## Results

### PET imaging and biodistribution of [^18^F]FEDAC

**Fig. 2:**
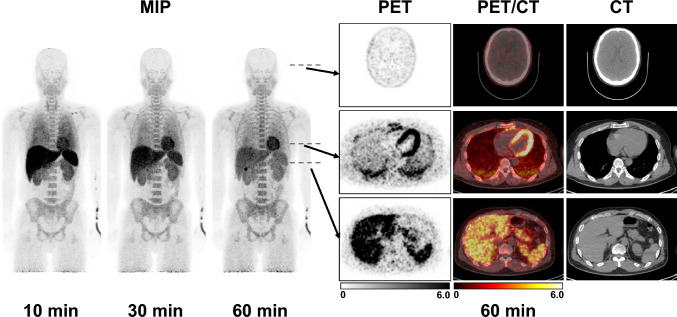
[^18^F]FEDAC-PET/CT Imaging in Normal Volunteer. Representative images (MIP images after 10 min, 30 min, and 60 min after injection and axial images of the head, chest, and upper abdomen.). The first 10-min image shows strong accumulation in the lungs, heart, liver, spleen, kidney, and bone marrow. Subsequently, the accumulation in the liver diminishes with time, and the radiotracer migration is seen from the bile duct to the gastrointestinal tract

Whole-body maximum-intensity projection images at 10, 30, and 60 min after the intravenous administration of [^18^F]FEDAC are shown in Fig. 2. The patterns of radiotracer accumulation showed no significant differences among the seven participants.

**Fig. 3:**
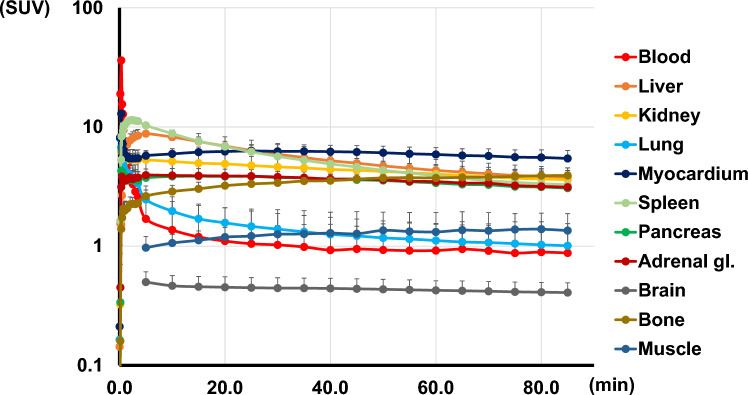
Time activity-curves of [^18^F]FEDAC in each organ. Time activity-curves are shown in logarithmic graph. There is a strong accumulation in the blood immediately after administration, which diminishes shortly afterwards. While most organs reach a plateau after 20 min, accumulation decreases with time in the spleen, liver, and lungs

The SUVmean for accumulation in the blood pool increased to 9.47 at 1 min, decreased to 1.69 at 5 min, and then decreased gradually. Strong accumulation was also observed in the liver immediately after intravenous injection, with SUVmean reaching 8.79 after 5 min, after which accumulation in the liver decreased and moved from the bile duct to the gastrointestinal tract. (Figs. 2 and 3, Table 1).

**Table 1:** Biodistribution and urine excretion of [18F]FEDAC in Normal volunteers. List of accumulation in each organ after injection. Urinary excretion was evaluated by % injected dose

Time (min)	1	3	5	10	20	30	40	60	80
Blood	9.47±1.52	3.32±0.23	1.69±0.25	1.37±0.25	1.10±0.24	1.02±0.25	0.93±0.25	0.92±0.25	0.89±0.27
Liver	4.79±0.80	8.06±1.05	8.79±0.99	8.23±1.10	6.91±1.36	5.91±1.38	5.21±1.25	4.32±1.02	3.86±0.81
Kidney	5.11±1.05	5.35±1.12	5.30±1.03	5.14±0.96	4.90±0.96	4.62±0.91	4.40±0.84	4.04±0.80	3.67±0.69
Lung	4.66±0.88	3.66±0.87	2.46±0.74	1.97±0.63	1.57±0.53	1.39±0.49	1.26±0.49	1.12±0.43	1.03±0.39
Myocardium	6.37±0.73	5.44±0.56	5.74±0.55	5.94±0.62	6.23±0.73	6.24±0.85	6.21±0.81	5.88±0.83	5.55±0.88
Spleen	9.80±1.95	11.42±1.88	10.33±1.82	8.80±1.83	6.85±1.55	5.67±1.25	4.90±1.07	3.91±0.71	3.35±0.52
Pancreas	3.98±0.73	3.58±0.62	3.75±0.70	3.84±0.64	3.84±0.59	3.76±0.48	3.61±0.42	3.39±0.33	3.14±0.31
Adrenals	3.55±0.79	3.65±1.07	3.94±1.19	3.92±1.11	3.88±1.04	3.81±0.90	3.66±0.91	3.49±0.82	3.17±0.78
Brain	n.d.	n.d.	0.50±0.11	0.47±0.10	0.45±0.10	0.44±0.10	0.44±0.10	0.43±0.09	0.41±0.09
Red Bone Marrow	2.06±0.29	2.27±0.28	2.63±0.34	2.87±0.37	3.22±0.45	3.40±0.41	3.54±0.45	3.73±0.54	3.91±0.57
Muscle	n.d.	n.d.	0.97±0.24	1.06±0.19	1.19±0.24	1.26±0.23	1.29±0.24	1.32±0.21	1.39±0.21

Time (min)	30	60	85
U.Bladder Content	0.06 (0.01–0.12)	0.13 (0.01–0.34)	0.19 (0.01–0.53)

[^18^F]FEDAC showed substantial accumulation in the myocardium, spleen, and kidneys. Moderate accumulation was observed in the lungs, adrenals, and red bone marrow. Despite the substantial accumulation in the kidneys (SUVmean > 5 at 1–5 min), urinary bladder accumulation after 60 min was 0.13% (0.01–0.34% injected dose (ID)). Radiotracer accumulation in the brain remained low from 85 min post-administration.

The SUVmean for accumulation in the red bone marrow was 2.63 at 5 min after administration; it gradually increased afterward and rose to 3.73 after 60 min.

### Radiation dosimetry

High absorbed doses were observed in the liver, gallbladder, kidney, pancreas, and spleen, with the highest absorbed dose in the ventricular wall. Doses of the significant organs per unit administration (μGy/MBq) and effective doses (mSv) assessed for the seven participants are shown in Table 2. The overall trend for the organ doses was similar among the participants. The range of administered activity was 201.8–218.0 MBq, and the variation of the effective doses was also minimal, i.e., 3.60 ± 0.15 mSv.

**Table 2:** Organ and Effective Doses for [^18^F]FEDAC PET Imaging estimated based on ICRP Publ. 103. Effective dose was calculated according to Publication 103 of the ICRP

Organs [μGy/MBq]	Sub. 1	Sub. 2	Sub. 3	Sub. 4	Sub. 5	Sub. 6	Sub. 7	Mean±s.d.
Adrenals	27.90	40.95	36.95	34.40	26.70	33.00	38.00	33.99±5.24
Brain	5.73	7.32	5.85	6.36	5.03	6.38	6.24	6.13±0.71
Breast	12.70	12.50	12.35	12.30	12.40	12.10	12.45	12.40±0.18
Colon Wall	13.55	13.75	13.65	13.90	13.65	14.25	13.80	13.79±0.23
Gallbladder wall	28.50	32.10	38.30	35.75	27.35	33.40	28.60	32.00±4.11
Heart wall	48.00	53.25	46.50	47.95	34.45	43.45	54.60	46.89±6.70
Kidneys	31.40	37.30	36.45	32.25	23.20	29.50	32.65	31.82±4.69
Liver	39.35	50.25	43.60	34.45	40.65	33.25	36.95	39.79±5.83
Lung	37.95	34.10	28.15	30.30	23.80	26.30	27.95	29.79±4.82
Muscle	11.60	11.09	11.55	11.75	12.25	11.95	11.85	11.72±0.36
Ovaries	15.60	15.00	15.50	16.20	16.30	16.70	16.00	15.90±0.57
Pancreas	29.35	36.35	36.15	31.50	26.60	31.00	34.95	32.27±3.69
Prostate	11.70	11.00	11.90	12.20	12.80	12.50	12.20	12.04±0.59
Red (active) Bone marrow	22.80	24.10	25.45	25.20	21.25	24.80	23.05	23.81±1.52
Salivary glands	17.25	23.95	20.75	21.45	14.30	19.80	24.25	20.25±3.56
Skin	8.79	8.44	8.69	8.92	9.23	9.10	8.97	8.87±0.26
Small intestine Wall	14.20	14.60	14.45	14.60	14.10	14.90	14.45	14.47±0.27
Spleen	32.20	35.70	31.20	28.95	24.00	23.85	32.85	29.82±4.50
Stomach wall	18.00	19.60	18.25	17.50	16.75	17.25	17.95	17.90±0.91
Testis	9.89	9.25	9.82	10.20	10.80	10.50	10.30	10.11±0.51
Thyroid	16.10	20.50	16.85	16.45	13.00	14.05	16.25	16.17±2.38
Ureters	15.05	15.00	16.00	15.75	15.75	15.75	15.60	15.56±0.38
Urinary bladder Wall	11.65	11.00	11.85	12.15	12.70	12.45	12.10	11.99±0.56
Uterine/Cervix	13.60	12.80	13.90	14.20	14.90	14.50	14.20	14.01±0.68
Effective dose [μSv/MBq]	20.20	21.10	19.80	19.50	17.70	18.70	19.30	19.47±1.08
Effective dose [mSv/185MBq]	3.74	3.90	3.66	3.61	3.27	3.46	3.57	3.60±0.20

### Safety evaluation

No adverse or clinically detectable pharmacological effects were observed in any of the seven participants. In addition, no significant changes were observed in vital signs or laboratory test results.

## Discussion

This study aimed to facilitate clinical imaging using [^18^F] FEDAC, a novel PET radiotracer for imaging the 18-kDa TSPO. This first-in-man study demonstrated the biodistribution of and the radiation exposure associated with [^18^F]FEDAC, which were similar to those of other agents reported to date, i.e., PK11195, PBR06, DAA1106, and FEDAA1106 [22–25]. The effective dose of [^18^F]FEDAC was 19.47 μSv/MBq, which was similar to that of [^18^F]FDG (24 μSv/MBq) and comparable to those of other ^18^F-labeled TSPO ligands (20.2 μSv/MBq for [^18^F] FEPPA and 18.5 μSv/MBq for ^18^F-PBR06), which also showed almost similar radiation exposure [23, 26]. The organ with the highest radiation exposure was the heart (46.89 µGy/MBq). The radiation dose for 185 MBq of FEDAC was 3.6 mSv, which was well below the standard of 10 mSv. Thus, clinical examinations with [^18^F]FEDAC can be performed more than once a year.

TSPO is highly expressed in the myocardium, kidneys, and adrenal glands, and FEDAC also showed substantial accumulation in these organs. The lungs also express high levels of TSPO, but radiotracer accumulation was relatively low because of the low tissue density. TSPO is expressed less in the liver; however, as with other tracers, substantial accumulation is observed in the liver immediately after injection of the radiotracer. Accumulation in the liver decreased with time, and the radiotracer was transferred from the bile to the digestive tract, suggesting that the liver is the principal eliminating organ of [^18^F]FEDAC. Unlike other agents, within the 90-min observation period after intravenous injection, the proportion of the radiotracer transferred into the urine was small, and no accumulation was observed in the skull, ribs, or long bones, indicating that the [^18^F]FEDAC was not defluorinated.

In preclinical studies using rats, the accumulation of radiotracers in the liver was weak, which is the most significant difference from the findings obtained in animal models. This may reflect the differences in metabolic pathways between animal models and humans. However, the strong accumulation in the ventricular wall and kidneys was consistent in human and animal models, which was considered to reflect the high expression of TSPO in tissues. The accumulation of [^18^F]FEDAC in the brain tissue was minimal.

Since this is the first-in-human study of [^18^F]FEDAC and the objective is to estimate safety and the biodistribution and radiation dose of the whole body, which required dynamic scan immediately after intravenous injection including the heart, the lungs, and the upper abdomen, followed by the whole-body static scans. Further studies are needed to evaluate the biodistribution of [^18^F]FEDAC in brain tissue to apply for neurological diseases in the future.

The biodistribution of [^18^F]FEDAC administered in this study varied slightly among the seven participants. Many second-generation TSPO-specific PET ligands have shown sensitivity variations associated with the RS6971 polymorphism. This genetic polymorphism results in large inter-individual variability in radioligand binding and is a significant obstacle to TSPO-PET imaging studies. To the best of our knowledge, the effect of the RS6971 polymorphism on [^11^C]DAC or [^18^F]FEDAC has not yet been reported. In this study, all seven participants showed high affinity for TSPO. According to the HAPMAP database, which is no longer publicly available, the proportion of patterns with no rs6971 mutations and high affinity in East Asian populations is 96.9%. Although the seven participants in our study were not genetically tested, they all probably showed an approximately similar pattern of accumulation because of their high affinity for the agent and the absence of genetic mutations. Additional studies on individuals with the RS6971 mutation are required.

## Conclusions

Radiation dosimetry for the TSPO imaging agent [^18^F]FEDAC was performed in this first-in-man study. The effective dose for the adult model was estimated as 19.47 μSV/ MBq, similar to that for [^18^F]-FDG. A diagnostic dose of 185 MBq for [^18^F] FEDAC is considered acceptable for its use as a diagnostic tool in TSPO imaging.

## Data Availability

The datasets generated and/or analyzed during the current study are available from the corresponding author on reasonable request.
